# Resilience of the larval slipper limpet *Crepidula onyx* to direct and indirect-diet effects of ocean acidification

**DOI:** 10.1038/s41598-017-12253-2

**Published:** 2017-09-21

**Authors:** Elizaldy A. Maboloc, Kit Yu Karen Chan

**Affiliations:** 1 School of Science, Hong Kong University of Science and Technology, Clear Water Bay, Kowloon, SAR Hong Kong; 2 Division of Life Science, Hong Kong University of Science and Technology, Clear Water Bay, Kowloon, SAR Hong Kong

## Abstract

Ocean acidification (OA) is known to directly impact larval physiology and development of many marine organisms. OA also affects the nutritional quality and palatability of algae, which are principal food sources for many types of planktonic larvae. This potential indirect effect of OA via trophic interactions, however, has not been fully explored. In this study, veligers of *Crepidula onyx* were exposed to different pH levels representing the ambient (as control) and low pH values (pH 7.7 and pH 7.3) for 14 days, and were fed with *Isochrysis galbana* cultured at these three respective pHs. pH, diet, nor their interactions had no effect on larval mortality. Decrease in pH alone had a significant effect on growth rate and shell size. Structural changes (increased porosity) in larval shells were also observed in the low pH treatments. Interactions between acidification and reduced diet quality promoted earlier settlement. Unlike other calcifying molluscs, this population of slipper limpets introduced to Hong Kong in 1960s appeared to be resilient to OA and decreased algal nutritional value. If this robustness observed in the laboratory applies to the field, competition with native invertebrates may intensify and this non-native snail could flourish in acidified coastal ecosystems.

## Introduction

Anthropogenic emission of carbon dioxide (CO_2_) to the atmosphere has been increasing and leads to the elevation of CO_2_ partial pressure (*p*CO_2_) in the ocean^1^. This process of ocean acidification (OA) has driven the average ocean pH to drop by 0.1 since pre-industrial times and is predicted to drive a further drop of 0.2–0.4 units by the turn of this century^1^^,^^2^. Continuing OA represents a major threat to a wide array of marine organisms^3^. Early life stages of marine invertebrates, especially calcifying larvae are particularly sensitive to OA^4–7^. Known effects of OA include reduced survivorship, changes in physiological processes, and decreased calcification rates^8–10^. Despite the overall negative impacts of OA, little is known about its indirect effects, including changes in ecological interactions^11^.

The potential effect of OA on food abundance and quality is one example of such important yet little studied ecological interactions. Food quality and quantity are known to affect survival, growth and larval competence of several marine invertebrates^12^^,^^13^. It is reported that OA was shown to alter the stoichiometry of algae^14–16^. Increase in *p*CO_2_ enhances inorganic carbon uptake^17^, which in turn increases the C:N ratio, and thus, decreases algal nutritional quality^14^^,^^18^^,^^19^. Elevated *p*CO_2_ can also reduce or alter the polyunsaturated fatty acids (PUFAs) composition of algae^20^, where PUFAs are critical for enzyme activity, stress resistance, growth and survival for various marine organisms^21^. These OA-induced changes in algal nutritional value was shown to affect feeding preference and growth rate of the amphipod *Orchestoidea tuberculata* ^16^. Whether this indirect, diet quality effect of OA translates to other species remains unknown. However, increased food quantity has been suggested to help ameliorate the negative impacts of increased *p*CO_2_^2^^,^^22^^,^^23^, and this effect was experimentally demonstrated in the mussel *Mytilus edulis* ^23^ and the barnacle *Amphibalanus improvisus* ^24^. These changes in algal abundance and nutritional content under OA condition may alter the herbivore’s feeding strategies and have yet to be fully investigated^16^.

Some invasive species were resilient to exposure to OA alone^25^^,^^26^. However, it is unclear whether the abovementioned changes in trophic interaction under future climate condition could negatively affect their ability to colonize and spread to new habitats. One such invasive species is *Crepidula fornicata*, their survivorship under acute exposure to high temperatures was unchanged regardless of rearing *p*CO_2_ levels(550, 750, 1,000 µatm)^27^. Increased *p*CO_2_ only negatively affected calcification in *C*. *fornicata* but did not affect the other physiological rates, e.g., respiration and ammonia excretion^26^. It is unclear whether its sister species,*Crepidula onyx,* which has invaded the South China coast^28^, shares the same level of resilience.

Food quality and quantity are known to affect larval development. Both *C*. *fornicata* and its sister species *C*. *onyx* have reduced growth rates at low food concentrations^29–33^. For both *Crepidula* spp., short-term starvation and nutritional stress during the larval stage reduced post-metamorphic growth in juveniles^29–32^. Larval growth of *C*. *fornicata* also varied with diet quality: larvae grew faster on a diet of *Isochrysis galbana*, which is rich in essential fatty acids^29^^,^^34^. Food quality and quantity are therefore critical in shaping the rate of larval development. Global climate change and ocean acidification are expected to alter both phytoplankton abundance and nutritional quality^29^, however, the interactive effects of OA and diet quality are still largely unknown. Here we exposed larvae of the slipper limpet, *C*. *onyx*, to acidified rearing conditions and/or algal diets and tested if this invasive species is robust towards OA stress and could continue to flourish in its non-native habitat.

## Results

### Seawater carbonate chemistry and algal C:N ratio

Seawater carbonate chemistry from the three experimental trials were pooled after an initial test showed no significant differences between trials (*p*CO_2_; Kruskal-Wallis test, *H*_2,42_ = 0.310, *p* = 0.856, Ω_Ar_; Kruskal-Wallis test, *H*_2,42_ = 0.938, *p* =0.626, and Ω_Ca_; Kruskal-Wallis test, *H*_2,42_ = 0.931, *p* = 0.628). For the larval rearing bottles, *p*CO_2_ (Kruskal-Wallis test, *H*_2,42_ = 35.880, *P* < 0.0001), Ω_Ar_ (Kruskal-Wallis test, *H*_2,42_ = 35.880, *P* < 0.0001) and Ω_Ca_ (Kruskal-Wallis test, *H*_2,42_ = 35.880, *P* < 0.0001) varied significantly between pH treatments (Table 1). Temperature between the three trials showed small and negligible (<0.2 °C) difference (Kruskal-Wallis test, *H*_2,42_ = 12.643, *p* = 0.002).

**Table 1:** Seawater carbonate chemistry parameters throughout the experiment. Seawater total scale pH, temperature and total mean alkalinity (mean TA: 2167.55 μmol.kg^−1^) were used to calculate CO_2_ partial pressure (*p*CO_2_), aragonite and calcite saturation states (respectively Ω_Ar_ and Ω_Ca_) for a salinity of 32.0 psu using the package seacarb for R. All the values are expressed as mean ± SD.

Larval rearing	Measured			Calculated		
	T (°C)	pH_T_	A_T_ (μmol/kg)	*p*CO_2_ (μatm)	Ω_Ar_	Ω_Ca_
Control pH + Control diet	23.44 ± 0.13^a^	8.04 ± 0.02	2155.71 ± 48.63	403 ± 19^a^	2.88 ± 0.10^a^	4.41 ± 0.15^a^
Medium pH + Control diet	23.28 ± 0.17^b^	7.71 ± 0.06	2141.35 ± 67.07	963 ± 140^b^	1.55 ± 0.15^b^	2.38 ± 0.23^b^
Low pH + Control diet	23.33 ± 0.05^b^	7.38 ± 0.04	2164.23 ± 40.11	1822 ± 89^c^	0.95 ± 0.04^c^	1.46 ± 0.06^c^
Control pH + Medium diet	23.48 ± 0.19^a^	7.99 ± 0.01	2165.19 ± 39.79	455 ± 13^a^	2.68 ± 0.06^a^	4.10 ± 0.10^a^
Control pH + Low diet	23.46 ± 0.16^a^	8.00 ± 0.02	2180.33 ± 23.75	441 ± 2^a^	2.72 ± 0.06^a^	4.17 ± 0.09^a^
Medium pH + Medium diet	23.27 ± 0.21^b^	7.71 ± 0.06	2174.60 ± 72.64	979 ± 123^b^	1.56 ± 0.16^b^	2.39 ± 0.25^b^
Low pH + Low diet	23.24 ± 0.14^b^	7.37 ± 0.03	2190.68 ± 29.08	2034 ± 162^c^	0.85 ± 0.11^c^	1.30 ± 0.17^c^

Values with different letters are significantly different from each other.

*p*CO_2_ (Kruskal-Wallis test, *H*_2,51_ = 44.4615, *P* < 0.0001), Ω_Ar_ (Kruskal-Wallis test, *H*_2,51_ = 44.1560, *P* < 0.0001) and Ω_Ca_ (Kruskal-Wallis test, *H*_2,51_ = 44.1560, *P* < 0.0001) saturation states also varied significantly between algal cultures (Table 2). Temperature between algal cultures showed no significant differences (Kruskal-Wallis test, *H*_2,51_ = 0.6865, *p* = 0.7095).

**Table 2:** Seawater carbonate chemistry parameters in the algal cultures and percent carbon and nitrogen content of *Isochrysis galbana* cultured at 3 different pH conditions. Seawater total scale pH, temperature and total mean alkalinity (mean TA: 2156.13 μmol.kg^−1^ were used to calculate CO_2_ partial pressure (*p*CO_2_), aragonite and calcite saturation states (respectively Ω_Ar_ and Ω_Ca_), for a salinity of 35.0 psu using the package seacarb for R. All values are expressed as mean ± SD.

Algal culture	Measured			Calculated			C:N ratio		
	T (°C)	pH_T_	A_T_ (μmol/kg)	*p*CO_2_ (μatm)	Ω_Ar_	Ω_Ca_	mg C g^−1^ DW	mg N g^−1^ DW	C:N
Control	27.64 ± 1.49^a^	9.15 ± 0.39	2172.78 ± 26.80	21 ± 35^a^	9.90 ± 1.55^a^	14.41 ± 2.34^a^	0.0367	0.0043	8.48^a^
Medium pH	27.42 ± 1.55^a^	7.74 ± 0.13	2154.06 ± 39.47	903 ± 266^b^	1.99 ± 0.62^b^	2.99 ± 0.93^b^	0.0464	0.0049	9.53^a^
Low pH	27.52 ± 1.49^a^	7.39 ± 0.11	2141.54 ± 37.30	2146 ± 573^c^	0.96 ± 0.24^c^	1.45 ± 0.36^c^	0.0490	0.0036	13.57^b^

Values with different letters are significantly different from each other.

Algae reared at different pH culture conditions had significantly different C:N ratios (ANOVA, *F*_2,3_ = 69.95, *p* = 0.003). Post hoc test showed that C:N ratio of the low pH culture condition (13.57) was significantly different from the control (Tukey’s test, *p* = 0.003) and from the medium pH (Tukey’s test, *p* = 0.006). There was no significant difference (Tukey’s test, *p* = 0.196) between the control with C:N ratio of 8.48 and medium pH culture condition with 9.53 (Table 2).

### pH and diet had no effect on the larval mortality and respiration rate

**Figure 1:**
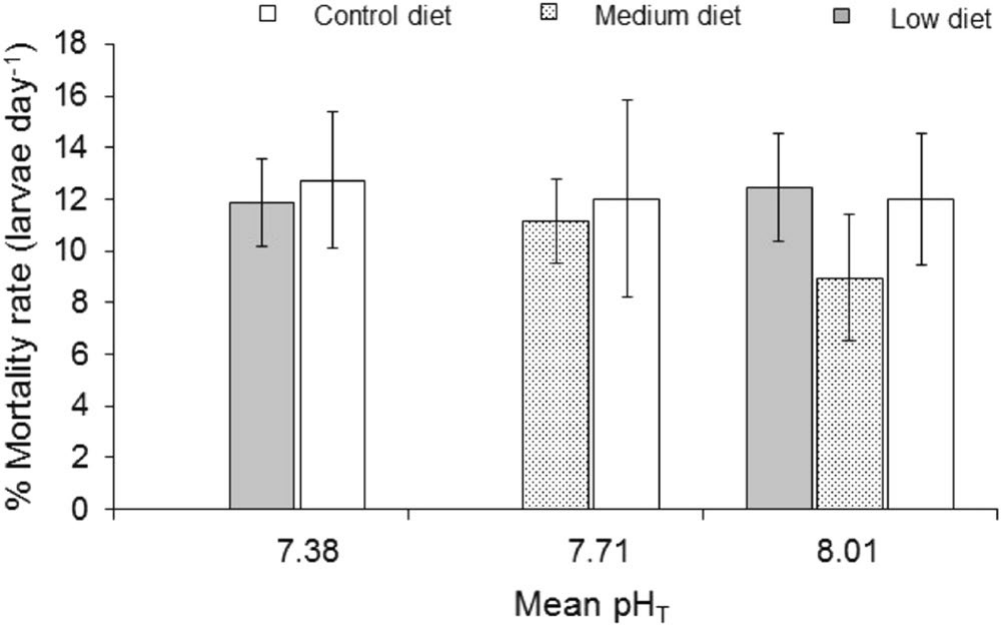
Larval mortality rate of *Crepidula onyx* was not affected by both pH and diet treatments. Error bars represent standard deviation (n = 6).

Larval mortality of *C*. *onyx* was not significantly affected by pH treatments (*p* = 0.552) nor diet (*p* = 0.272, Fig. 1, Table 3). In addition, pH and diet interactions had no effect on mortality (*p* = 0.534). Respiration rates (nmol O_2_ hr^−1^ μm BL^−1^) were not significantly affected by pH treatments, diet and pH and diet interactions (Fig. S1). However, sampling days showed significant effects on the respiration rates i.e. higher oxygen consumption rates in older larvae (Fig. S1).

**Table 3:** Results of the analysis of variance (ANOVA) for all larval traits measured in *Crepidula onyx*.

Parameters	Source of variation	d.f.	*F*-value	*p*-value
Mortality rate	pH	2	0.61	0.55
	Diet	2	1.35	0.27
	pH x diet	2	0.64	0.53
Shell length	pH	2	11.78	0.0001*
	Diet	2	0.78	0.47
	pH x diet	2	1.15	0.33
Shell area	pH	2	7.90	0.006*
Growth rate	pH	2	3.30	0.048*
	Diet	2	2.08	0.14
	pH x diet	2	1.14	0.33
Settlement	pH	2	1.84	0.21
	Diet	2	3.08	0.10
	pH x diet	2	6.58	0.02*
Clearance rate	pH	2	2.40	0.11
	Diet	2	284.68	<0.0001*
	pH x diet	4	2.26	0.09

*Significant results (P < 0.05).

### pH affected larval shell length, growth rate and shell integrity

**Figure 2:**
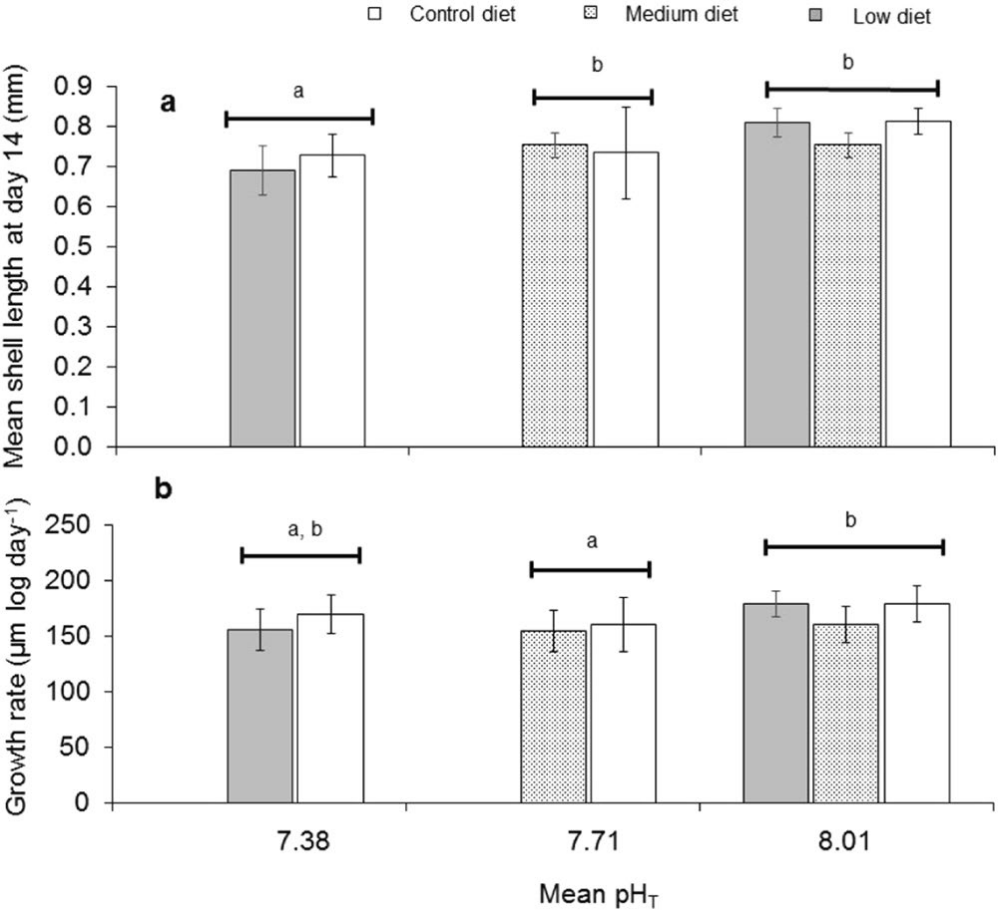
Mean shell length at day 14 (**a**) and growth rate (**b**) of *Crepidula onyx* exposed to different pH levels and diets. pH had significant effects on both shell length and growth rate while diet nor the pH and diet interactions showed no significant effects. Due to uneven number of larvae measured, mean of means of shell length was used. Bar graphs with different letters are significantly different from each other. Error bars represent standard deviation (n = 6).

Shell length (SL) on day 14 was significantly affected by pH treatments (*p* = 0.0001, Fig. 2a, Table 3). Post hoc test showed that larvae in the low pH treatment were significantly smaller the other two treatments (Tukey’s test, *p* < 0.0006). Newly settled juveniles on day 14 had a mean SL of 0.79 ± 0.04 mm in the control, 0.74 ± 0.8 mm in medium pH and 0.71 ± 0.06 mm in the low pH. However, diet had no significant effect on SL (*p* = 0.468), nor did the pH and diet interactions (*p* = 0.329). Larval shell area on day 14 also differed significantly between pH treatments (*p* = 0.007), ranging from 0.014 ± 0.002 mm^2^ in the control pH to 0.010 ± 0.002 mm^2^ in the low pH treatment. Growth rate (Fig. 2b) was also significantly affected by pH treatments (*p* = 0.048) and growth rate in the control was significantly higher than those in medium pH treatments (Tukey’s test, *p* = 0.019). Mean growth rate was 173.09 ± 16.60 µm log day^-1^ in the control, 157. 48 ± 20.69 µm log day^-1^ in the medium pH and 162.85 ± 18.47 µm log day^-1^ in the low pH treatment. Diet had no significant effect on the growth rate (*p* = 0.139), nor did the interactions between pH and diet (*p* = 0.330).

**Figure 3:**
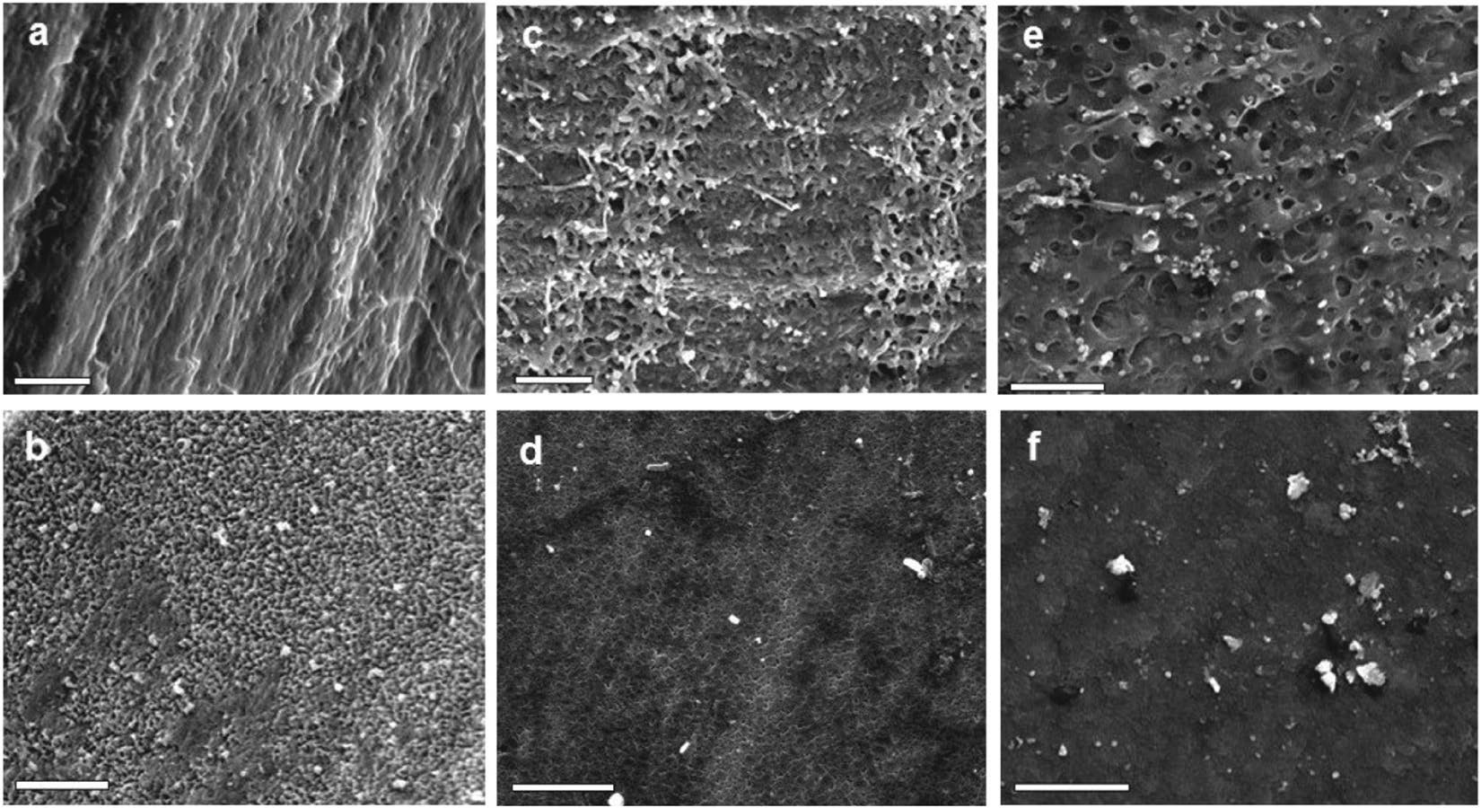
Scanning electron micrographs showing the ultrastructures of day 14 *Crepidula onyx* shells exposed to control pH (**a**,**b**), medium pH (**c**,**d**) and low pH (**e**,**f**). *Scale bars* = 5 *µm*.

Results from scanning electron microscopy (Fig. 3) revealed minor but noticeable structural changes in the larval shells at low pH conditions. The growing outer edge of the nacreous layers differed between pH treatments: under control pH conditions, the shell exhibited clear nacreous layers (Fig. 3a) and a defined crystallites, granulated structures (Fig. 3b); medium pH resulted in perforated nacreous layers (Fig. 3c) and slightly eroded periostracum layers (Fig. 3d), while low pH treatment led to nacreous layers with pits and perforations (Fig. 3e), and heavily eroded crystallite structures in the periostracum layer (Fig. 3f).

### pH and low diet quality enhanced larval settlement

**Figure 4:**
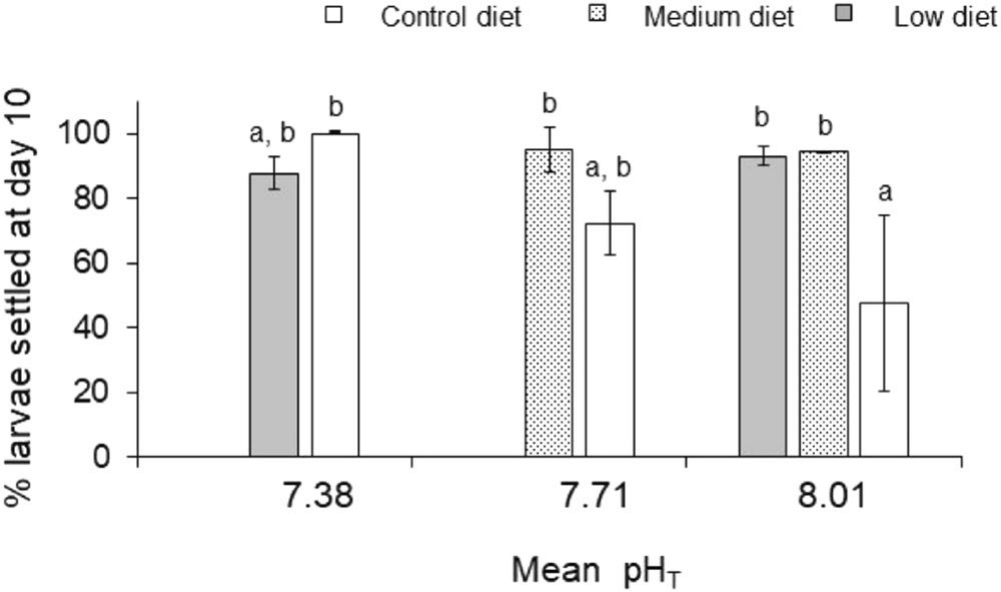
Percent spontaneously settled larval *Crepidula onyx* after being exposed to different pH levels and diets for ten days. No significant difference in the number of settled larvae on day 10 between pH and diet; but significant interactions between pH and diet was observed and letters indicate the post-hoc Tukey’s test grouping. Error bars represent standard deviation (n = 6).

The percentage of larvae settled was not affected by pH (*p* = 0.372) nor diet (*p* = 0.367) alone, but was affected by pH and diet interactions (*p* = 0.017). It appeared that larval settlement was enhanced with decreasing pH in combination with low diet quality (Tukey’s test, *p* < 0.05, Fig. 4).

### Low diet quality enhanced clearance rate

**Figure 5:**
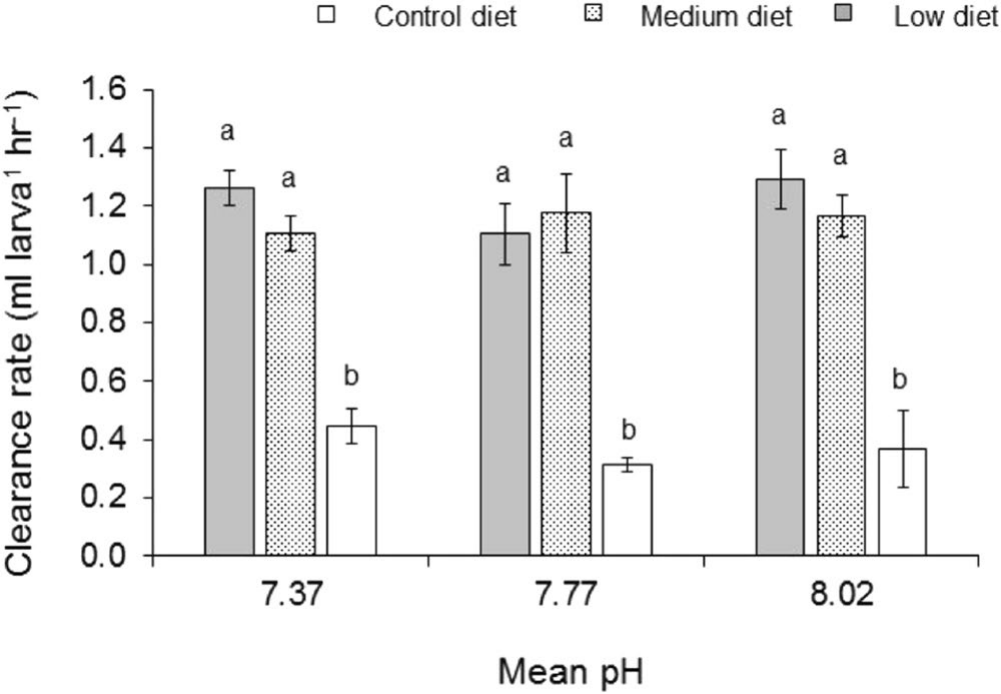
Larval clearance rate of *Crepidula onyx* estimated through incubating of known amount of larvae with known amount of algal cell for 2 hours. Significant differences in clearance rates were observed between diet qualities, with high clearance rates at low diet quality. Bar graphs with different letters are significantly different from each other. Error bars represent standard deviation (n = 4).

Clearance rate (CR) of *C*. *onyx* was not significantly affected by pH treatments (*p* = 0.109) but was significantly affected by diet (*p* < 0.0001, Fig. 5, Table 3). Clearance rates were significantly lower in the control diet than the other two lower diets (Tukey’s test, *p* < 0.001). However, pH and diet interactions had no effect on the CR (*p* = 0.089).

## Discussions

Not only does ocean acidification impact larval physiology, it can also affect larval performance through indirect interactions, including the change in algal-prey nutritional value as OA reduces the nutritional quality of marine algae by elevating the C:N ratio. However, this reduction in food quality together with reduced pH (pH 7.3) did not affect the mortality and respiration rate of larval *Crepidula onyx*. Decrease in pH alone reduced *C*. *onyx* larval shell length, growth rate and shell structure. Low pH and low diet quality, however, appeared to promote larval settlement. Our work suggests that some species, including *C*. *onyx*, exhibit plasticity to cope with, if not are already well adapted to ocean acidification and low algal nutritional value.

The decrease in pH alone had a significant effect on shell size (10% decrease), growth rate (13% decrease), and shell structures. As diet did not affect shell length and growth rate, ultrastructure changes were assumed to be an effect due to pH alone. Similarly, larval shell deformities were observed in *C*. *fornicata* at high *p*CO_2_ (1400 µatm) condition, approximately pH_T_ 7.56^25^ and in *Mytilus edilus* at increasing *p*CO_2_ (1,120, 2,400 and 4,000 µatm)^35^. These changes in shell structures could imply a reduced calcification rate^36^ and/or increased dissolution. Future studies using radioactively labelled isotopes could help differentiate between these two processes. Reduction in biocalcification could indicate an energetic tradeoff ^37^ as maintaining homeostasis is energetically costly, e.g., ion transport accounted for over 80% energy expenditure in larval urchin exposed to OA conditions^38^. The ecological consequence of such tradeoff, e.g., increased vulnerability to predator^39^, warrants additional studies.

Despite pH effects on shell growth and morphology, larval mortality rate and respiration of *C*. *onyx* were not affected by acidification. Contrary to other species that showed metabolic depression under OA conditions, e.g., *Littorina littorea* ^40^ and *Mytilus chilensis* ^41^, *C*. *onyx* together with *C*. *fornicata* experienced little pH impact on their respiration rate. Such resilience could be attributed on several factors. These factors include: 1) pre-exposure to low *p*CO_2_ condition in their habitats and inside brood chamber. Coastal environments in Hong Kong can experience seasonal low pH as low as 7.7 pH unit^42^^,^^43^. *Crepidula onyx* brood their larvae in brood chambers for 2 to 4 weeks prior to release, and pH therein could be low, e.g., pH 7.0 in *Ostrea chilensis* ^42^ and 6.4 in the calyptraeid *Crepipatella dilatata* ^44^; 2) presence of a large energy reserve from yolk, e.g., Gallager and Mann^45^ showed that survival of bivalve larvae positively correlated with lipid content in eggs and varied between broodstock. The Hong Kong population has relatively larger egg size (mean size of 181.75 µm) compared to the populations from California (172 µm)^46^ and Panama (157–159 µm)^47^. Further, starved larvae from this local population were able to settle though at significantly smaller sizes, which would suggests *C*. *onyx* larvae have relatively high energy reserve (Maboloc and Chan, pers. obs.); 3) presence of maternal transferred protein, e.g., larval oysters of *Crassostrea sikamea* collected from polluted sites are more resistant to trace metal stress due to maternal transferred metallothionein^48^; 4) high efficiency ion transport through energetic trade-offs and or energy re-allocation (see above discussion); 5) changes in feeding behaviors (see below); or a combination of the factors above.

Low pH and low diet quality promoted larval settlement. Dooley and Pires^49^ reported a similar observation for *C*. *fornicata*, where larvae settled and metamorphosed at higher frequency at lower pH (pH 7.5 and pH 7.7) than the control (pH 8.0). Plasticity in settlement schedule could be an indication of larval stress or reduction of energy reserve as postulated by the “desperate larva hypothesis”^50^^,^^51^. Assuming little to no difference in post-settlement mortality this increase in settlement will likely have a positive implication on the number of individuals recruiting to the population^52^. Alternatively, these earlier settlers could also suffer higher mortality as they could be less selective against poor settlement sites^50^. Together with the lack of impact on larval mortality, OA and low algal nutritional value likely have little overall impact on the local population of *C*. *onyx* in Hong Kong.

Diet quality alone has no significant effect on the larval development of *C*. *onyx*. Larvae could have met its metabolic requirements by increasing its feeding in response to low food quality as the food concentration was non-limiting in this study. Results from supplementary feeding experiments conducted with larvae from one of the females studied showed that larvae exposed to reduced pH and/or fed with low-pH grown algae, increased their clearance rates by three fold when compared to those in the control (Fig. 5, Table 3). Similar increase in clearance rates with the increasing *p*CO_2_ was also observed in *C*. *fornicata* ^26^. Blue mussel *Mytilus edulis* larvae also showed high feeding rates at pH 7.35^53^. This observed increase in clearance rate could potentially incur energetic cost and affect energy allocation. However, of the small number of marine invertebrate studied, ciliary motion for both swimming (e.g., 0.5–1.5% larval energy stores hr^−1^ in *Bugula* spp.)^54^ and feeding (e.g., 0.502 calories day^−1^ in *Menippe mercenaria*)^55^ accounted only a small portion of their energy budget. It is however possible that under a food limiting condition, acidification-induced reduction in food quality could have negative impacts.

If the high individual resilience of *C*. *onyx* observed in the laboratory is realized as transgenerational plasticity in the field, this invasive species may have competitive advantage over the local species, which might eventually lead to shifts in community compositions under future ocean conditions. However, to fully understand invasive dynamics of *C*. *onyx*, further studies are needed to identify the impact of more realistic multiple stressors scenario and how long term exposure to these environmental changes affects its reproductive success, settlement and dispersal.

## Materials and Methods

### Adult collection and broodstock maintenance

Slipper limpets *Crepidula onyx* were collected from Victoria Harbor, Hong Kong (22°29′N, 114°17′E). Both adults and larvae acquired were reared to sexual maturity under laboratory conditions at the Coastal Marine Laboratory, Hong Kong University of Science and Technology (~7 months to first brooding). The animals were maintained in filtered (0.2 μm) seawater (pH_T_ = 8.09 ± 0.10; T = 22 °C; S = 32; light/dark cycle: 12 h/12 h) with water changes every other day and with daily feeding of *Isochrysis galbana* at 4 × 10^5^ cells ml^−1^. Since adult slipper limpet *C*. *fornicata* can consume and digest larvae and zooplankton^56^. To add more nutrients, *C*. *onyx* adults were supplemented with newly hatched *Artemia* nauplii at ~30 individual ml^−1^ twice a week. After ~7 months of rearing, egg capsules were being brooded and veligers were released into the water. The swimming veligers from each individual were collected immediately after their release through a 100 μm sieve and counted for use in subsequent experiments.

### Experimental design, larval rearing, and seawater carbonate chemistry

To test the direct and diet-mediated indirect effects of ocean acidification on the development of *C*. *onyx*, the larvae were exposed to three pH levels (control pH ≈ 8.01, medium pH ≈ 7.71 and low pH ≈ 7.38) and fed with algae cultured at different pH, hereinafter referred to as diet, which had different nutritional qualities as indicated by C:N ratio (*see algal culturing*). These pH levels were chosen to represent both present day extreme values in the coastal environments (pH_NBS_ 7.7–8.2 in Hong Kong^42^^,^^43^ and the native habitat of *C*. *onyx* where upwelling occurs (with values < pH 7.75)^57^, as well as the predicted surface pH reduction of 1.4 units by 2300^58^.

A total of 7 treatments was tested: namely larvae reared at control pH and fed with algae grown in high, medium, and low pH (treatments 1–3), larvae reared at medium pH fed with algae grown in control and medium pH (treatments 4–5), and larvae reared at low pH fed with algae grown in control and low pH (treatments 6–7). There were duplicate rearing bottles for each treatment during each experimental trial. The experiment was repeated three times with larvae from different mothers. Each of the mothers was kept with two males.

Larvae were reared in 1.5 l filtered seawater at a density of 1 larva 5 ml^−1^ (~300 larvae per bottle) and maintained at a temperature of 23 ± 2 °C. Culture bottles were cleaned and water was completely changed with pre-equilibrated CO_2_ filtered seawater on day 4, day 8, and day 12 post hatching. The experiment was terminated on day 14. Larvae were fed every day (4 × 10^5^ cells ml^−1^) starting from day 0 with *I*. *galbana* cultured at 3 different pH conditions (*see algal culturing*).This ad libitum concentration was chosen based on Zhao *et al*.^33^ as low food concentration alone negatively affect larval growth and development. To check if food addition can cause pH variations in the rearing bottles, pH was measured once before and after food addition. pH variations were minimal and within the experimental levels (e.g., before food addition, pH value at one low pH bottle was 7.361, after adding control food, pH value was 7.425). More importantly, all cultures were continuously aerated with a gas mixture at the experimental *p*CO_2_ level, pH deviations stabilized quickly to the set level.

Each larval culture was continuously aerated and mixed through gentle air bubbling. The pH in the medium and low cultures were controlled by constant addition of a mix of compressed air and pure CO_2_ controlled by a thermal mass flow controller (GFC 17 Aalborg, New York USA; ±1% FS accuracy). The pH, millivolt, and temperature of each culture bottle were monitored daily with Metrohm 826 pH meter and Unitrode (Herisau, Switzerland). Salinity was measured using a handheld refractometer. The pH was converted to the total scale (pH_T_) after calibration with TRIS (Tris/HCl) buffer solution with a salinity of 33.0 provided by the Dickson Lab at the Scripps Oceanographic Institute. Duplicate samples for total alkalinity (TA) were taken on day 4, day 8, day 12 and day 14 from all the cultures and from the newly filtered, pH equilibrated seawater used to refill the jars (n = 3). A computer-driven titration system (905 Titrando mounted with a glass electrode; Unitrode with Pt 1000; Herisau, Switzerland) was use to assess the TA of filtered samples (0.2 μm) with a Gran function, as described by Dickson *et al*.^59^. The carbonate system parameters (*p*CO_2_, Ω_Ar_ and Ω_Ca_) were calculated from these two measurements with the R package seacarb^60^ using the dissociation constants from Mehrbach *et al*.^61^ as refitted by Dickson and Millero^62^.

### Algal culturing

*Isochrysis galbana* was sub-cultured from the same algal starter and maintained in f/2 medium under 3 different pH_T_ conditions (9.15 ± 0.39 as control/regular culture method, medium pH_T_ 7.74 ± 0.13, and low pH_T_ 7.39 ± 0.11). To achieve the medium and low pH levels, algal cultures were bubbled with pure CO_2_ controlled by pH-stat systems (R-WP017 CO_2_ Regulator, Easy-Aqua, Guangzhou, China). pH level in the control raised from the ~pH 8.0 of ambient seawater to ~ pH 9.0 as the algae photosynthesize. Cell density was determined by collecting aliquot samples (n = 3) and each were counted three times with a hemacytometer. pH_T,_ temperature, and salinity were measured daily. Algal cultures at exponential phase (4–5 days) were used to feed the larvae. To test for differences in food quality between the cultures, 2 sub-samples of 100 ml from the 3 culture conditions were filtered onto pre-combusted Whatman GF/F glass filters and processed for carbon to nitrogen ratio (C:N) with 2400 Series II CHNS/O Elemental Analyzer (Perkin Elmer, MA, USA) following the protocol of USEPA standard method 440.0^63^.

### Larval development, shell growth and settlement

Larval cultures were sampled every other day starting from day 1 until day 14 (50 ml subsample × 2) to assess larval density. Samples were immediately fixed with a drop of buffered formalin solution (4% in FSW at pH 8.3), veliger larvae were counted and stored in 2% buffered formalin solution at 4 °C until further measurements. Water volume in each culture jar was adjusted at every water change and sampling to maintain the initial density of the culture. For each culture, survival (S) was calculated for each day as the proportion of larval density divided by the maximum number of larvae counted during the experiment. Mortality rates (MR) were computed as the coefficient of significant linear regression between survival and time (% larvae day^−1^, Table S1).

At least five larvae (8 ± 2 larvae per sampling point and treatments) from the fixed samples were photographed under the microscope (Nikon H600L, Japan) and shell length (SL) was measured with ImageJ^64^. Growth rates (GR) were calculated for each culture as the regression coefficient of the significant logarithmic relationship between measured SL and time (Table S2). A subsample from each of the cultures was also collected on day 3, day 7 and day 14 for larval respiration measurements (*supplementary method*).

Nine days post hatching, three subsamples of 20 larvae from each of the duplicate rearing bottles in each treatment were collected and placed in six-well plates with 5 ml of respective pre-equilibrated CO_2_ to assess spontaneous settlement (n = 6). After 24-hours, the number of larvae swimming and settled or attached were counted.

### Shell integrity and larval shell area

For shell integrity and morphology, a subsample of fixed larvae from each treatment (n = 5) were collected on day 14 and rinsed three times in phosphate buffer saline, cleared through a gradient of ethanol until 100% concentration and freeze dried. The samples were then mounted on a stub, viewed and photographed with scanning electron microscope (JEOL JSM - 6390 MA, USA) at the Materials Characterization and Preparation Facility at HKUST. Larval shell area was then measured with ImageJ.

### Clearance rate

Maternal half-sibling larvae from one brood were collected from the 3 different pH cultures (control pH ≈ 8.02, medium pH ≈ 7.77 and low pH ≈ 7.37). In each feeding experiment, 5 *C*. *onyx* larvae were placed into a 50 ml falcon tube with 25 ml pre-equilibrated pH treated seawater. The larvae were acclimated for an hour before feeding and placed onto a custom made plankton rack for mixing. For each treatments, larvae were fed with 2 × 10^5^ cells ml^−1^ of *Isochrysis galbana* cultured at 3 respective pHs. The treatments and blank procedural controls (without the larvae) were incubated for 2 hours then fixed with 4% buffered formalin. The algal concentration before and after the experiment was enumerated with a Beckman Z2 Coulter^®^ Particle Count and Size Analyzer (California, USA). The clearance rate following the formula of Ginger *et al*.^65^ was calculated from the decrease in the algal concentration during the 2-hour feeding period {[(ln B_1_ − ln B_0_) − (ln C_1_ − ln C_0_) V]/t}/n; B_1_ = blank control, initial algal concentration, B_0_ = blank control, final algal concentration, C_1_ = treatment, initial algal concentration, C_0_ = treatment, final algal concentration, V = 25 ml, t = 2 h, n = 5 larvae and expressed in milliliters per larva per hour (ml larva^−1^ h^−1^). There were 4 replicates per pH and diet treatment.

### Data analyses

All the statistical analyses were carried out with the software Statistica 7 and significance levels of α = 5%. All data were checked for normality (Shapiro-Wilk test) and homogeneity (Levene’s test). Data from all the experiments were pooled after an initial test (Univariate test) showed no significant differences between the three experimental trials. Two-way Analyses of Variance (ANOVA) were used to analyze effects of pH treatments and diet on mortality rate, growth rate, larval shell area, settlement, clearance rate, temperature, and C:N ratio. Three-way ANOVA was used to analyze the respiration rates (log transformed). Post-hoc Tukey tests were performed when significant differences were detected. Seawater carbonate chemistry was analyzed with the non-parametric Kruskal-Wallis test.

## Electronic supplementary material

Supplementary Material
