# Supplementary material for: Resilience of the larval slipper limpet Crepidula onyx to direct and indirect-diet effects of ocean acidification
Source: Sci Rep. 2017 Sep 21;7:12062. doi: 10.1038/s41598-017-12253-2 (PMC5608699; doi:10.1038/s41598-017-12253-2)
Supplement: Supplementary file 1 — Supplementary Material [file 41598_2017_12253_MOESM1_ESM.doc]

**Resilience of the larval slipper limpet *Crepidula onyx* to direct and indirect-diet effects of ocean acidification**

**Elizaldy A. Maboloc^1^ and Kit Yu Karen Chan^2,*^**

^1^ School of Science, Hong Kong University of Science and Technology, Clear Water Bay,

Kowloon, Hong Kong, SAR

Tel. + (852) 6024-7165, email: eamaboloc@connect.ust.hk

^2^ Division of Life Science, Hong Kong University of Science and Technology, Clear Water Bay,

Kowloon, Hong Kong, SAR

Tel. + (852) 2358-7998, email: karenchan@ust.hk

**Supplementary material**

Materials and methods

*Larval respiration*

A subsample from each of the cultures was collected on day 3, day 7 and day 14 for larval respiration measurements. Oxygen consumption rates were measured with micro plate readers (Loligo PreSens, Netherlands) equipped with oxygen sensors integrated with a 24 channel pre-calibrated fluorescence-based respirometry system (SDR sensor dish). Larvae were hand-picked under a stereomicroscope, thoroughly rinsed with filtered (0.2 μm) seawater at the designated pH. These individuals were then randomly assigned to one of twenty four 200 μl micro-wells (n = 5 larvae per well, 2 wells per culture bottle). The seawater used in rinsing and incubation was previously filtered (0.2 μm), fully oxygenated and equilibrated to the appropriate pH. Respiration due to potential bacterial contamination was evaluated in wells devoid of larvae (2 wells per pH level and per micro-plate). Each well was then sealed with the PET caps provided by the manufactures, and the micro-well plates were placed in a temperature-regulated water bath (22.87 ± 0.34°C). Oxygen concentration was measured in each well every 15 to 30 seconds and was monitored for at least 6 hours. Respiration rates were calculated with a linear regressions between oxygen concentrations in each well and time after removing initial noise (Chan *et al.,* in review). The estimated rate was then corrected by the number of larvae per well, the average measured size of the larvae in each culture (O_2_ consumption expressed as μmol O_2_ hr^-1^ larva^-1^ μm^-1^), as well as the respiration in the bacterial controls.

**Table S1** Larval mortality rates (MR) were calculated as the coefficient of the significant relationship between survival and time (% larvae day^-1^).

| **Larval rearing** | **Female** | **Trial** | **R** | **MR (% larvae day^-1^)** | ***p - value*** | ***r^2^*** | ***F-value*** | ***df*** |
| --- | --- | --- | --- | --- | --- | --- | --- | --- |
| Control pH + Control diet | 1 | 1 | 1 | 15.25 | 0.032 | 0.83 | 14.33 | 4 |
|  |  |  | 2 | 10.40 | 0.049 | 0.66 | 7.80 | 5 |
|  | 2 | 2 | 1 | 13.67 | 0.0001 | 0.97 | 164.28 | 6 |
|  |  |  | 2 | 10.71 | 0.014 | 0.73 | 13.49 | 6 |
|  | 1 | 3 | 1 | 8.47 | 0.027 | 0.74 | 11.56 | 5 |
|  |  |  | 2 | 13.42 | 0.0001 | 0.96 | 121.98 | 6 |
| Medium pH + Control diet | 1 | 1 | 1 | 16.92 | 0.006 | 0.88 | 28.71 | 5 |
|  |  |  | 2 | 10.14 | 0.025 | 0.67 | 10.07 | 6 |
|  | 2 | 2 | 1 | 12.50 | 0.006 | 0.81 | 21.19 | 6 |
|  |  |  | 2 | 9.23 | 0.028 | 0.74 | 11.31 | 5 |
|  | 1 | 3 | 1 | 7.34 | 0.013 | 0.82 | 18.23 | 5 |
|  |  |  | 2 | 15.93 | 0.001 | 0.89 | 42.05 | 6 |
| Low pH + Control diet | 1 | 1 | 1 | 10.85 | 0.013 | 0.74 | 14.42 | 6 |
|  |  |  | 2 | 12.96 | 0.031 | 0.83 | 14.79 | 4 |
|  | 2 | 2 | 1 | 10.08 | 0.017 | 0.71 | 12.41 | 6 |
|  |  |  | 2 | 12.12 | 0.042 | 0.8 | 11.72 | 4 |
|  | 1 | 3 | 1 | 17.64 | 0.021 | 0.77 | 13.73 | 5 |
|  |  |  | 2 | 12.78 | 0.029 | 0.64 | 9.06 | 6 |
| Control pH + Medium diet | 1 | 1 | 1 | 6.80 | 0.049 | 0.57 | 6.72 | 6 |
|  |  |  | 2 | 10.40 | 0.032 | 0.72 | 10.39 | 5 |
|  | 2 | 2 | 1 | 10.13 | 0.0003 | 0.97 | 143.72 | 5 |
|  |  |  | 2 | 5.07 | 0.024 | 0.76 | 12.55 | 5 |
|  | 1 | 3 | 1 | 10.96 | 0.044 | 0.59 | 7.12 | 6 |
|  |  |  | 2 | 10.42 | 0.041 | 0.60 | 7.52 | 6 |
| Control pH + Low diet | 1 | 1 | 1 | 11.81 | 0.001 | 0.98 | 98.61 | 3 |
|  |  |  | 2 | 13.71 | 0.046 | 0.91 | 20.16 | 3 |
|  | 2 | 2 | 1 | 10.05 | 0.007 | 0.79 | 18.93 | 6 |
|  |  |  | 2 | 10.71 | 0.0002 | 0.95 | 86.54 | 6 |
|  | 1 | 3 | 1 | 15.69 | 0.024 | 0.86 | 18.2 | 4 |
|  |  |  | 2 | 12.70 | 0.017 | 0.71 | 12.37 | 6 |
| Medium pH + Medium diet | 1 | 1 | 1 | 12.08 | 0.002 | 0.99 | 504.1 | 3 |
|  |  |  | 2 | 11.04 | 0.046 | 0.67 | 8.06 | 5 |
|  | 2 | 2 | 1 | 10.95 | 0.014 | 0.73 | 13.83 | 6 |
|  |  |  | 2 | 13.59 | 0.002 | 0.98 | 128.37 | 4 |
|  | 1 | 3 | 1 | 10.58 | 0.015 | 0.89 | 25.56 | 4 |
|  |  |  | 2 | 8.64 | 0.037 | 0.70 | 9.52 | 5 |
| Low pH + Low diet | 1 | 1 | 1 | 9.82 | 0.027 | 0.66 | 9.53 | 6 |
|  |  |  | 2 | 14.50 | 0.013 | 0.98 | 78.37 | 3 |
|  | 2 | 2 | 1 | 11.56 | 0.02 | 0.69 | 11.26 | 6 |
|  |  |  | 2 | 12.35 | 0.005 | 0.82 | 23.23 | 6 |
|  | 1 | 3 | 1 | 10.30 | 0.048 | 0.58 | 6.79 | 6 |
|  |  |  | 2 | 12.74 | 0.026 | 0.66 | 9.79 | 6 |

**Table S2** Larval growth rate was derived from the coefficient of logarithmic regression of mean shell lengths against time.

| **Larval rearing** | **Female** | **Trial** | **R** | **GR (um log day**^-1^**)** | ***p - value*** | ***r^2^*** | ***F-value*** | ***df*** |
| --- | --- | --- | --- | --- | --- | --- | --- | --- |
| Control pH + Control diet | 1 | 1 | 1 | 210.46 | 0.008 | 0.79 | 18.35 | 6 |
|  |  |  | 2 | 177.02 | 0.007 | 0.79 | 18.94 | 6 |
|  | 2 | 2 | 1 | 164.97 | 0.014 | 0.73 | 13.78 | 6 |
|  |  |  | 2 | 171.96 | 0.013 | 0.74 | 14.09 | 6 |
|  | 1 | 3 | 1 | 180.42 | 0.002 | 0.87 | 34.69 | 6 |
|  |  |  | 2 | 170.28 | 0.003 | 0.85 | 29.15 | 6 |
| Medium pH + Control diet | 1 | 1 | 1 | 168.23 | 0.009 | 0.77 | 16.28 | 6 |
|  |  |  | 2 | 141.84 | 0.012 | 0.75 | 15.06 | 6 |
|  | 2 | 2 | 1 | 191.05 | 0.020 | 0.78 | 13.88 | 5 |
|  |  |  | 2 | 122.81 | 0.005 | 0.82 | 21.97 | 6 |
|  | 1 | 3 | 1 | 164.64 | 0.017 | 0.71 | 12.29 | 6 |
|  |  |  | 2 | 173.23 | 0.001 | 0.91 | 53.44 | 6 |
| Low pH + Control diet | 1 | 1 | 1 | 159.51 | 0.008 | 0.78 | 18.04 | 6 |
|  |  |  | 2 | 189.44 | 0.009 | 0.78 | 17.52 | 6 |
|  | 2 | 2 | 1 | 192.61 | 0.009 | 0.77 | 16.41 | 6 |
|  |  |  | 2 | 159.47 | 0.006 | 0.80 | 20.54 | 6 |
|  | 1 | 3 | 1 | 149.69 | 0.013 | 0.74 | 14.47 | 6 |
|  |  |  | 2 | 168.08 | 0.016 | 0.72 | 12.87 | 6 |
| Control pH + Medium diet | 1 | 1 | 1 | 143.09 | 0.015 | 0.72 | 13.08 | 6 |
|  |  |  | 2 | 144.94 | 0.001 | 0.96 | 86.22 | 5 |
|  | 2 | 2 | 1 | 173.24 | 0.012 | 0.75 | 14.98 | 6 |
|  |  |  | 2 | 151.14 | 0.039 | 0.61 | 7.71 | 6 |
|  | 1 | 3 | 1 | 180.94 | 0.005 | 0.82 | 22.83 | 6 |
|  |  |  | 2 | 170.92 | 0.004 | 0.84 | 25.77 | 6 |
| Control pH + Low diet | 1 | 1 | 1 | 179.70 | 0.009 | 0.77 | 16.80 | 6 |
|  |  |  | 2 | 192.44 | 0.006 | 0.80 | 20.14 | 6 |
|  | 2 | 2 | 1 | 160.39 | 0.006 | 0.80 | 20.42 | 6 |
|  |  |  | 2 | 190.14 | 0.003 | 0.85 | 27.56 | 6 |
|  | 1 | 3 | 1 | 178.34 | 0.003 | 0.85 | 29.18 | 6 |
|  |  |  | 2 | 175.25 | 0.003 | 0.84 | 27.15 | 6 |
| Medium pH + Medium diet | 1 | 1 | 1 | 167.13 | 0.004 | 0.83 | 24.14 | 6 |
|  |  |  | 2 | 165.70 | 0.008 | 0.79 | 18.64 | 6 |
|  | 2 | 2 | 1 | 145.35 | 0.001 | 0.90 | 46.00 | 6 |
|  |  |  | 2 | 121.13 | 0.021 | 0.69 | 11.14 | 6 |
|  | 1 | 3 | 1 | 164.09 | 0.0002 | 0.95 | 85.39 | 6 |
|  |  |  | 2 | 164.52 | 0.005 | 0.89 | 33.05 | 5 |
| Low pH + Low diet | 1 | 1 | 1 | 170.04 | 0.004 | 0.84 | 26.95 | 6 |
|  |  |  | 2 | 164.83 | 0.004 | 0.83 | 24.82 | 6 |
|  | 2 | 2 | 1 | 121.56 | 0.006 | 0.80 | 20.43 | 6 |
|  |  |  | 2 | 149.80 | 0.006 | 0.81 | 21.05 | 6 |
|  | 1 | 3 | 1 | 165.25 | 0.004 | 0.90 | 34.45 | 5 |
|  |  |  | 2 | 163.93 | 0.018 | 0.79 | 14.98 | 5 |


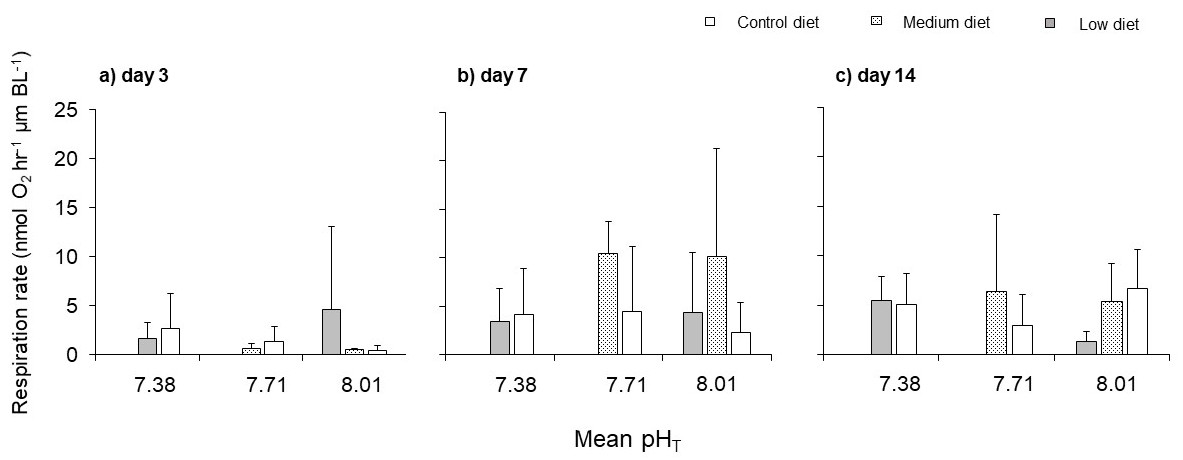


**Fig. S1** Respiration rates of *Crepidula onyx* measured on day 3 (a), day 7 (b) and day 14 (c). Error bars represent standard deviation (n = 6). Respiration rates (nmol O_2_ hr^-1^ μm BL^-1^) were not significantly affected by pH treatments (ANOVA, *F*_2, 77_ = 1.435, *P* = 0.245). Diet also did not affect the respiration rates (ANOVA, *F*_2, 77_ = 1.479, *P* = 0.234). However, respiration rates were significantly different between days (ANOVA, *F*_2, 77_ = 86.491, *P* < 0.0001). Significant differences (Tukey’s test, *P* = 0.021) were observed between all sampling days (Tukey’s test, *P* < 0.01). pH and diet interactions (ANOVA, *F*_2, 63_ = 0.077, *P* = 0.926) and pH, diet and days interactions (ANOVA, *F*_4, 63_ = 1.429, *P* = 0.2345) showed no significant effects in the respiration rates.
